# Hydrastinine and Calcium Chloride

**Published:** 1894-02-03

**Authors:** 


### NEW DRUGS AND PREPARATIONS.

[We shall be glad to receive, at our Office, 428, Strand, London, W.C., from the manufacturers, specimens of all new preparations and drugs which may be brought out from time to time.]

### HYDRASTININE AND CALCIUM CHLORIDE.

By D. R. PATERSON, M.D., M.R.C.P., Hon. Pathologist Cardiff Infirmary.

Any remedy that is serviceable in the arrest of internal hæmorrhage will always find a welcome from the practitioner. Two of the more recent, hydrastinine hydrochlorate and calcium chloride are now on trial, and it may help to bring them into greater prominence and secure an extended experience, which is so necessary for a final judgment, if a few notes concerning them are furnished here. In their pharmacological actions they represent the methods adopted by nature in the spontaneous arrest of bleeding, viz., contraction of the vessel wall and coagulation of the blood, and we owe their introduction to laborious research.

Hydrastinine, or, to speak more correctly, hydrochlorate of hydrastinine, is derived from hydrastine by oxidation, the latter being an alkaloid found in the rhizome of *Hydrastis canadensis.* A tincture of this plant had been long in use as a stomachic tonic, and it is only recently on the discovery of hydrastinine and researches into its nature that attention has been called to its action in the control of bleeding. Naturally its administration has been largely directed towards that great source of hæmorrhage—the uterus, and in this respect it falls at once into comparison with another most valuable drug—ergot of rye. Hydrastinine is said to act on the blood vessel wall, causing constriction without stimulating the unstriped muscular fibres of the uterus as ergot does. Whether it can be proved to do so is uncertain, but it may be accepted in explanation of a difference in action between the two substances, as pointed out by experience. We would gather from this that where contraction of the uterine muscular fibre is essential or conducive to the stoppage of hæmorrhage, hydrastinine is of little use, or much inferior to ergot, and as a matter of fact we do not find it of much value in post-partum flooding or the bleeding associated with myoma. These hæmorrhages are much more satisfactorily treated by ergot, which possesses the power of inducing contraction not only in the unstriped muscle of the uterus, but also in that of the blood vessels, which are thus constricted and compressed as well. On the other hand, where congestive changes in the pelvic organs are the source of untoward hæmorrhage, periodical or otherwise, we find hydrastinine to be more suitable than ergot. To this category belongs the great class of menorrhagias, and it is in this direction that we hope to obtain scope for the therapeutical application of this drug. It is necessary to discriminate at the outset between the varieties of menorrhagia, for where it is dependent on local morbid conditions, such as gross disease of the uterus, it is obvious local measures must be adopted. Where such processes can be excluded, and it is probable the hæmorrhage is conditioned by pelvic vascular engorgement, hydrastinine is of great service. Accordingly it has been administered in the menorrhagia of young women and of the menopause, and in the form associated with disease of the appendages, where the uterus is involved in the congestive changes. The success met with justifies the praise bestowed, for in it I believe we have a remedy which is a useful adjunct in dealing with these affections so troublesome to the practitioner, menorrhagia and congestive dysmenorrhœa. The form of administration may be the liquid extract of Hydrastis canadensis given in twenty or thirty drop doses three times a day. It is, however, somewhat uncertain, and hydrastinine itself is more reliable. This substance should not be confounded with the alkaloid hydrastine, as the former, although derived from it by oxidation, is much more powerful in its chemical and physiological action. The best form for the exhibition of hydrastinine is by capsule or "perle," one half to three-quarters of a grain four or five times a day. In this way the dosage is more accurate; the bitter taste may be avoided and the drug is better borne by the stomach.

Calcium chloride is so well known a substance that one might be almost inclined to question its classification as a "new remedy." Such is, however, the case, for it is but very recently that the muriate of lime has been suspected of possessing the power of arresting internal hæmorrhage. For this knowledge we are indebted to those painstaking researches on the coagulability of the blood, one of the fruits of which is revealed in this unexpected quarter. Professor Wright, of Netley, to whom we owe much of what is known of this matter, says "appropriate additions of lime salts increase the rate and firmness of coagulation in the diluted (blood) plasma" (*Journal of Pathology*, June, 1893). In another paper he records that he found from observations on his own blood, by a method which is applicable to clinical purposes, that it clotted in thirty seconds with the addition of a lime salt, when its usual coagulation time was represented by two and a quarter minutes. Of still greater interest are experiments performed on a man suffering from hæmophilia where there is a tardiness in coagulation that makes even trifling bleeding difficult to stop. To this patient was given a dose of fifteen grains of calcium chloride three times a day, with the result that the coagulation period was reduced from ten minutes to half that time. This effect is maintained, however, only up to a certain point, and further administration of the same quantity of lime salt not only does not shorten the period but actually increases it. Experiments on normal blood confirm this, and Professor Wright believes that the greatest accelerations of coagulation time (*i.e.*, increase of coagulability) are obtained with comparatively small doses of lime. On his own person "the effect of taking thirty grains of calcium chloride in a single dose was a reduction of coagulation time from a normal of four minutes to one and three-quarter minutes, twenty-four hours after the exhibition of the lime." The importance of these facts can hardly be overrated, for the action furnishes a means of strengthening the methods employed by nature in the arrest of hæmorrhage. Apart from hæmophilia, where it will be found especially useful, internal bleeding from different organs is indicated for treatment with good hopes of success; and for aneurism, where it is desirable to promote a natural cure in the direction of clotting, we are put in possession of a valuable aid. Of all the remedies employed in the control of hæmorrhage none exhibits the power of assisting coagulation to the same degree as muriate of lime. Moreover, it may be used to advantage in combination with other drugs, such as ergot and gallic acid, the *modus operandi* of which lies in a different pharmacological action. The point to be borne in mind in its administration is to avoid large doses, a maximum quantity beyond which it is unsafe to go ought to be fixed. It is better here to err on the side of doing too little, and a dose of five grains three times a day is sufficient. Saundby uses the liquor calcis chloridi (1 to 5) of the Pharmacopœia, and never gives more than thirty minims or six grains. Where a rapid effect is desired, a large dose of thirty grains may be administered; but in cases where the object is rather to prevent or ward off hæmorrhage the quantity mentioned above should not be exceeded.

